# An SPP1-centered immune–parenchymal communication axis links macrophage heterogeneity to ovarian cancer prognosis

**DOI:** 10.3389/fimmu.2026.1949243

**Published:** 2026-09-22

**Authors:** Xiangyu Liu, Li Xia, Jindong Sheng, Xuchen Cao, Ke Wang

**Affiliations:** 1 Department of Gynaecological Oncology, Tianjin Medical University Cancer Institute and Hospital, National Clinical Research Center for Cancer, Key Laboratory of Cancer Prevention and Therapy, Tianjin’s Clinical Research Center for Cancer, Tianjin, China; 2 The First Department of Breast Cancer, Tianjin Medical University Cancer Institute and Hospital; National Clinical Research Center for Cancer, Key Laboratory of Cancer Prevention and Therapy, Tianjin’s Clinical Research Center for Cancer, Tianjin, China; 3 Department of Breast, Sichuan Clinical Research Center for Cancer, Sichuan Cancer Hospital & Institute, Sichuan Cancer Center, University of Electronic Science and Technology of China, Chengdu, China

**Keywords:** cell – cell communication, ovarian cancer, prognostic model, single-cell RNA sequencing, SPP1, tumor-associated macrophages

## Abstract

**Background:**

Immune and malignant epithelial cells shape each other through ligand–receptor signaling in ovarian cancer. SPP1 interacts with CD44 and several integrins, but its cellular organization and clinical relevance remain unclear.

**Methods:**

We integrated GSE184880 scRNA-seq data with TCGA-OV and three independent cohorts. Single-cell clustering, CNV inference, and cell communication analysis were used to characterize macrophage states and SPP1-related signaling. Based on the SPP1 ligand–receptor network identified by CellChat, an 11-gene set comprising SPP1 and its CD44/integrin receptor components was constructed and subsequently used for molecular subtyping. Four machine learning models were compared for prognostic prediction. PI3 was subsequently silenced in ES-2 and SKOV3 cells. SPP1 expression and secretion, cell proliferation, wound closure, migration, invasion, xenograft growth, and macrophage phenotypes after coculture were then evaluated.

**Results:**

Four macrophage states were identified: Resident Macro, Inflammatory Macro, SPP1+ TAM, and Activated Macro. Cell communication was extensively reorganized in ovarian tumors. SPP1+ TAM occupied a central position in the SPP1 network and communicated with malignant epithelial cells, fibroblasts, endothelial cells, and other macrophage populations through SPP1–CD44 and SPP1–integrin interactions. SPP1-related genes separated patients into SPP1-High and SPP1-Low subtypes. The SPP1-High subtype showed stronger immune- and cytokine-related activity and poorer overall survival. Among the four machine learning models, GBM–Cox achieved the highest C-index of 0.768 and maintained prognostic stratification across three independent cohorts. PI3 also ranked highly in several models. Silencing PI3 in tumor cells increased TNF, IL1B, CXCL10, and CD86 expression in cocultured macrophages, while reducing CD163, MRC1, CCL18, and IL10. Consistently, TNF-α and IL-12p70 levels increased, whereas IL-10 and CCL18 levels decreased. PI3 silencing reduced SPP1 mRNA expression and secretion and suppressed ovarian cancer cell proliferation, wound closure, migration, and invasion. Stable PI3 knockdown also delayed ES-2 xenograft growth and reduced terminal tumor volume and weight.

**Conclusion:**

The SPP1-associated ligand–receptor network represents an immune–parenchymal communication axis linking macrophages with malignant epithelial and stromal cells in ovarian cancer. This network captures differences in immune state and clinical outcome and supports both molecular classification and prognostic assessment. PI3 may contribute to tumor cell-mediated regulation of macrophage phenotype and could serve as a candidate biomarker or intervention target. PI3 may promote ovarian cancer progression while contributing to tumor-cell-derived SPP1 production and macrophage regulation, supporting its potential value as a candidate biomarker and therapeutic target.

## 1 Introduction

Cells within a tissue microenvironment do not function in isolation. Through soluble mediators, extracellular matrix components, and direct or indirect ligand–receptor interactions, immune cells continually influence the behavior of parenchymal cells (1, 2). In tumors, these signals not only regulate immune-cell recruitment and activity but also affect cancer-cell proliferation, migration, metabolism, and stress adaptation (3). Compared with a simple description of immune infiltration, defining specific immune–parenchymal ligand–receptor axes may provide a clearer view of how the microenvironment shapes disease-associated cellular phenotypes (4).

Ovarian cancer is characterized by marked microenvironmental heterogeneity (5). Malignant epithelial cells coexist with macrophages, lymphocytes, fibroblasts, and endothelial cells in a densely connected cellular network (6). Tumor cells can alter immune states through chemokines, inflammatory mediators, and matrix-related signals, while immune cells can in turn influence tumor invasion, survival, and treatment response (7). Because the same signal may act on several cell populations, its biological relevance cannot be fully understood from expression level alone. The cellular source of a ligand, the distribution of its receptors, and the direction of communication may all affect the resulting tissue state.

Macrophages occupy a central position between immune activity and the phenotype of tumor parenchymal cells (8). The conventional M1/M2 framework does not adequately reflect the continuous and diverse states adopted by tumor-associated macrophages (9). Single-cell studies have identified inflammatory, tissue-resident, proliferative, and SPP1-expressing macrophage populations within tumors (10). These populations differ in the signals they produce and receive and may contribute differently to inflammation, extracellular matrix remodeling, angiogenesis, and immune suppression. Examining the ligand–receptor relationships between macrophages, malignant epithelial cells, and other tissue populations may therefore offer a useful route to understanding microenvironmental regulation in ovarian cancer.

SPP1 is a secreted protein with both cytokine-like and extracellular matrix-related properties. It binds CD44 and several integrin receptors (11). Through these receptors, SPP1 can participate in cell adhesion, migration, matrix remodeling, and immune regulation, while linking macrophages with tumor cells, fibroblasts, and endothelial cells (12). SPP1-expressing macrophages have been reported in multiple malignancies and are frequently associated with immunosuppressive features and unfavorable clinical outcomes (13). SPP1–CD44 and SPP1–integrin interactions may therefore form a multicellular microenvironmental axis that connects immune-cell states with tumor parenchymal phenotypes (14, 15). In ovarian cancer, however, the cellular organization, receptor distribution, and clinical significance of this network remain incompletely understood.

scRNA-seq and computational communication analysis now make it possible to investigate immune–parenchymal signaling at cellular resolution (16). By identifying ligand-producing cells, receptor-expressing populations, and the direction of signaling, these approaches can reconstruct relationships that are obscured in bulk tissue profiles. Integrating single-cell communication patterns with bulk transcriptomic data, independent cohorts, and clinical outcomes may further reveal whether a local signaling network reflects patient-level molecular states and supports prognostic stratification (17). Computational inference alone, however, cannot establish the functional role of a candidate gene, making experimental validation an important part of this process.

In this study, we investigated immune–parenchymal communication in ovarian cancer by integrating the GSE184880 scRNA-seq dataset, TCGA-OV, and several independent validation cohorts. We first characterized cellular composition, macrophage heterogeneity, and communication networks in nonmalignant ovarian tissues and HGSOC tissues, with particular attention to the distribution of SPP1-related ligand–receptor interactions among macrophages, malignant epithelial cells, and stromal populations. An SPP1-related gene set was subsequently constructed from the SPP1 ligand–receptor network identified by CellChat and was fixed before the downstream bulk-transcriptomic analyses. This gene set was then used to define molecular subtypes, which were compared in terms of survival, immune composition, and pathway activity. Multiple machine learning approaches were further applied to develop a prognostic model. Because PI3 showed high prognostic importance during model construction, PI3 was silenced in ovarian cancer cells to evaluate its effects on SPP1 expression and secretion, tumor-cell proliferation, migration and invasion, xenograft growth, and macrophage phenotype after coculture. By linking ligand–receptor communication, patient risk stratification, and functional validation, this study provides a cellular framework for understanding how the ovarian cancer microenvironment regulates disease-associated cell states.

## 2 Materials and methods

### 2.1 Data sources and study design

The scRNA-seq dataset GSE184880 was obtained from the GEO database. It comprised 12 ovarian tissue samples, including tumor tissues from seven treatment-naïve patients with high-grade serous ovarian cancer (HGSOC) and five age-matched nonmalignant ovarian tissue samples. Expression matrices and sample annotations were used to construct the single-cell atlas, characterize macrophage subsets, and infer cell–cell communication. Transcriptomic and clinical data from the TCGA-OV cohort were used for SPP1-related classification, prognostic gene screening, and model training. GSE140082, GSE26712, and GSE13876 were used as independent validation cohorts. Samples lacking survival time, survival status, or expression data for the model genes were excluded. Following CellChat analysis of the single-cell dataset, an SPP1-related gene set was constructed from the ligand and receptor components involved in the inferred SPP1 communication network. The resulting gene set was then fixed and applied to the TCGA-OV cohort for molecular subtype identification. Survival, pathway activity, and immune features were then compared between the subtypes. A machine learning-based prognostic model was subsequently developed. Single-cell data were further used to trace the cellular origin of SPP1 signaling and examine the role of macrophages in this network. Finally, coculture experiments were performed to evaluate the effect of PI3 in ovarian cancer cells on macrophage phenotype.

### 2.2 SPP1-related classification and survival analysis

The SPP1-related gene set was not obtained from a predefined external signature. It was constructed from the SPP1 ligand–receptor network identified by CellChat in the GSE184880 single-cell dataset. Specifically, the gene set included the ligand SPP1 and the receptor components involved in the identified SPP1–CD44 and SPP1–integrin interactions, namely CD44, ITGAV, ITGB1, ITGB3, ITGB5, ITGB6, ITGA4, ITGA5, ITGA8, and ITGA9. The resulting 11-gene set was fixed before consensus clustering and subsequent survival-related analyses in the TCGA-OV cohort. No survival or prognostic information was used during gene-set construction. The expression of these genes in the TCGA-OV cohort was used for consensus clustering. Repeated subsampling was performed to assess clustering stability across different cluster numbers. The optimal number of clusters was selected according to the consensus matrix, cumulative distribution function curves, and changes in the area under the CDF curve. Patients were finally assigned to the SPP1-High or SPP1-Low subtype. SPP1 pathway activity was quantified using gene set scoring. Principal component analysis was used to examine transcriptional differences between the two subtypes. Overall survival was compared using Kaplan–Meier analysis and the log-rank test. Hazard ratios and 95% confidence intervals were estimated using Cox regression.

### 2.3 Subtype-related genes and functional states

Differential expression analysis was performed between the SPP1-High and SPP1-Low subtypes. Significant genes were subjected to GO and KEGG enrichment analyses. Genes with marked expression differences were displayed in a heatmap. Pathway activity was also calculated at the individual-sample level to avoid relying only on differentially expressed genes. GO and KEGG pathway scores were compared between the two subtypes using linear models. The analysis focused on immune responses, cytokine signaling, cell cycle regulation, DNA repair, metabolism, and ciliary movement. Subtype-related genes were further assessed by univariate Cox regression. Genes with hazard ratios above 1 were considered risk-associated. Those with hazard ratios below 1 were considered protective. Genes with P < 0.05 were retained for machine learning analysis.

### 2.4 Immune cell infiltration analysis

The immune composition of TCGA-OV samples was estimated using CIBERSORT (18). The LM22 signature matrix was used to infer the relative proportions of 22 immune cell types. Only samples with a CIBERSORT output P value below 0.05 were retained. Immune cell proportions were compared between the SPP1-High and SPP1-Low subtypes using the Wilcoxon rank-sum test. This analysis was used to determine whether the two SPP1 states were associated with different immune microenvironments.

### 2.5 Prognostic model development and algorithm comparison

The TCGA-OV cohort was used for model training. Candidate genes identified by univariate Cox analysis were entered into four machine learning models: LASSO–Cox, GBM–Cox, random survival forest, and ABESS–Cox. For LASSO–Cox, the penalty parameter was selected by cross-validation. For GBM–Cox, the number of iterations was determined according to the cross-validation error. Variable importance was used to estimate the contribution of each gene. The stability of the random survival forest was evaluated using the out-of-bag error. ABESS–Cox was applied to obtain a more compact set of features. The four models were compared using the concordance index. Bootstrap resampling was used to assess the stability of the C-index estimates. GBM–Cox achieved the highest C-index and was selected as the final model. Its predicted relative risk was used as the patient risk score.

### 2.6 Model validation and risk stratification

The final model was evaluated in TCGA-OV, GSE140082, GSE26712, and GSE13876. Expression data were standardized separately within each cohort before risk scores were calculated. Patients were divided into high- and low-risk groups using the median risk score in each cohort. Overall survival was compared using Kaplan–Meier curves and the log-rank test. Time-dependent ROC curves were generated to assess the prediction of 1-, 3-, and 5-year survival. Because follow-up was limited in GSE140082, only the 1- and 3-year ROC curves were evaluated. Principal component analysis based on the model genes was used to visualize the distribution of the two risk groups. The same model structure was applied to all cohorts, whereas the median cutoff was determined separately within each dataset.

### 2.7 Single-cell data processing and major cell type annotation

A separate single-cell object was created for each sample in GSE184880. The downloaded 10X expression matrices contained 65,820 cell barcodes before study-specific quality control. Cells were retained when they had 200–7,500 detected genes, 500–60,000 total RNA counts, and a mitochondrial transcript proportion of no more than 30%. After quality control, 54,701 cells were retained for downstream analysis. The data were then normalized, and highly variable genes were selected. Scaling and principal component analysis were then performed. Harmony (19) was used to reduce sample-related batch effects, with sample identity specified as the correction variable. A nearest-neighbor graph was constructed from the corrected low-dimensional space. Unsupervised clustering and UMAP visualization were then performed. Cluster-specific genes were identified by differential expression analysis. Cell identities were assigned using canonical markers and cluster-specific expression patterns. The major populations included epithelial cells, T cells, NK cells, B cells, plasma cells, macrophages, endothelial cells, and fibroblasts. The proportion of each cell type was calculated for every sample. Differences in cellular composition between nonmalignant ovarian tissues and HGSOC tissues were assessed at the sample level.

### 2.8 Identification of malignant epithelial cells

All epithelial cells were extracted for the inference of large-scale copy number alterations. Epithelial cells derived from nonmalignant ovarian tissues were used as the reference population. Genes were ordered by chromosomal position to visualize broad gains and losses. The inferred CNV matrix was subjected to K-means clustering. A CNV score was calculated for each cell according to its deviation from the reference expression level.

The cluster with the lowest CNV signal and a profile close to that of the reference cells was defined as normal epithelial cells. The remaining clusters showed higher CNV scores and broader chromosomal alterations and were therefore classified as malignant epithelial cells. These malignant cells were labeled as tumor cells in the communication analysis.

### 2.9 Macrophage subtypes and functional annotation

Macrophages were extracted from the integrated single-cell object and independently normalized, reduced, and reclustered into eight subclusters. Based on an integrated assessment of cluster-level transcriptional profiles, representative marker genes, and functional enrichment patterns, these subclusters were subsequently grouped into four higher-order macrophage states: Resident Macro, Inflammatory Macro, SPP1+ TAM, and Activated Macro. Resident Macro was characterized by genes such as C1QC, FOLR2, and MRC1, whereas Inflammatory Macro mainly expressed FCN1, S100A8, and IL1B. SPP1+ TAM was marked by SPP1, GPNMB, and APOC1. The term Activated Macro was used as an operational, higher-order annotation encompassing macrophages with prominent proliferative and cell-cycle activity, together with cells showing interferon-responsive activation features. MKI67 and CXCL10/ISG15 were therefore considered representative markers of partially overlapping activation programs rather than a conjunctive marker combination required to be expressed by every cell. This annotation was not intended to define a transcriptionally homogeneous terminal macrophage subtype. The proportion of each macrophage state was calculated for every sample and compared between nonmalignant ovarian tissues and HGSOC tissues. State-specific genes were further analyzed using GO, KEGG, and Hallmark gene sets to characterize their principal functional programs.

### 2.10 Cell–cell communication and SPP1 network analysis

Cell communication networks were inferred separately for nonmalignant ovarian tissues and HGSOC tissues (20). After harmonizing the cell-type annotations between groups, the final CellChat objects included 24,299 cells from five nonmalignant ovarian tissue samples and 26,973 cells from seven HGSOC tissue samples, comprising 11 shared cell populations. NK cells were excluded because they were detected only in the nonmalignant group. Interactions involving cell populations represented by fewer than 10 cells were excluded using the min.cells = 10 filter. Normalized expression data and cell-type annotations were used as input, and human ligand–receptor interactions were used as the signaling reference. Overexpressed ligands and receptors were first identified for each cell group. Communication probabilities between cell populations were calculated with population.size = FALSE, meaning that cell-population size was not used as an explicit weighting factor. Individual ligand–receptor interactions were further summarized at the signaling pathway level. Nonmalignant ovarian and HGSOC tissues were compared in terms of interaction number, overall communication strength, and incoming and outgoing signaling activity. Increased and decreased interactions in HGSOC tissues were analyzed separately. For macrophage-centered analysis, the four macrophage subtypes were defined as signal senders or receivers. Their interactions with tumor cells, T cells, B cells, plasma cells, endothelial cells, and fibroblasts were examined. The SPP1 pathway was extracted from the global communication network. Hierarchical and circular plots were used to display signal sources, receiving populations, and communication strength. SPP1–CD44 and SPP1–integrin interactions were further compared between nonmalignant ovarian and HGSOC tissues. Detailed cell numbers and relative proportions for each CellChat object are provided in **Supplementary Table 1**.

### 2.11 Cell culture and PI3 knockdown

The human ovarian cancer cell lines ES-2 and SKOV3 and the human monocytic cell line THP-1 were maintained in culture medium supplemented with fetal bovine serum and antibiotics. Cells were cultured at 37 °C in a humidified atmosphere containing 5% CO_2_. For the *in vitro* experiments, two independent siRNAs targeting PI3 were used, and a nonspecific siRNA was used as the negative control. PI3 expression was measured by qRT-PCR after transfection to confirm knockdown efficiency. For the *in vivo* experiment, ES-2 cells stably expressing a PI3-targeting shRNA or the corresponding negative-control shRNA were established. The target sequence of sh-PI3 was 5′-GATCCCGTTAAAGGACAAGTT-3′ (TRCN0000073667).

### 2.12 Macrophage induction and Transwell coculture

THP-1 cells were seeded in the lower chamber of six-well plates at 5 × 10^5^ cells per well and treated with 100 ng/mL PMA for 24 h to induce adherence and differentiation into M0-like macrophages. The PMA-containing medium was then replaced with fresh PMA-free medium, and the cells were allowed to recover for an additional 24 h. An indirect coculture system was established using 0.4-μm Transwell inserts. ES-2 or SKOV3 cells transfected with si-NC or PI3-targeting siRNA were seeded in the upper chamber at 2 × 10^5^ cells per insert. The cells were cocultured at 37 °C in a humidified atmosphere containing 5% CO_2_ for 48 h. The M0 group contained macrophages cultured without tumor cells, whereas the si-NC and si-PI3 groups contained macrophages cocultured with control-transfected and PI3-silenced tumor cells, respectively. Macrophages and culture supernatants from the lower chamber were collected after coculture for qRT-PCR and ELISA analyses.

### 2.13 qRT-PCR and cytokine measurement

Total RNA was extracted from ovarian cancer cells or macrophages collected after coculture. After reverse transcription, relative gene expression was measured by qRT-PCR and calculated using the 2^−ΔΔCt^ method. GAPDH was used as the internal control. PI3 expression was measured in ovarian cancer cells to confirm knockdown efficiency. SPP1 mRNA expression was also examined in ovarian cancer cells after PI3 knockdown. The sequences of all primers used for qRT-PCR are provided in **Supplementary Table 2**. To measure tumor-cell-derived SPP1, the culture medium was replaced with fresh medium after transfection, and conditioned medium was collected after an additional 24 h. Cellular debris was removed by centrifugation, and secreted SPP1 was quantified using a human SPP1 ELISA kit according to the manufacturer’s instructions. SPP1 concentrations were normalized to the number of viable cells in the corresponding culture wells. In cocultured macrophages, the M1-related genes TNF, IL1B, CXCL10, and CD86 and the M2-related genes CD163, MRC1, CCL18, and IL10 were measured by qRT-PCR. The levels of TNF-α, IL-12p70, IL-10, and CCL18 in the culture supernatants were measured by ELISA. All cell experiments were independently repeated three times. All qRT-PCR and ELISA experiments were performed using three independent biological replicates, with each replicate derived from independently cultured and treated cells.

### 2.14 Cell proliferation, wound-healing, migration, and invasion assays

Cell proliferation following PI3 knockdown was evaluated using the CCK-8 assay. ES-2 and SKOV3 cells transfected with si-NC, siPI3-1, or siPI3–2 were seeded into 96-well plates, and absorbance at 450 nm was measured at 0, 24, 48, 72, and 96 h. For the wound-healing assay, a linear scratch was generated after the cells had formed a confluent monolayer. Images were obtained at 0 and 24 h, and the wound-closure rate was calculated. Cell migration and invasion were evaluated using uncoated and Matrigel-coated Transwell inserts, respectively. Cells that migrated or invaded through the membrane were fixed, stained, and counted in representative microscopic fields. All experiments were independently repeated three times.

### 2.15 Subcutaneous xenograft model

Five- to six-week-old female BALB/c nude mice were randomly allocated to the sh-NC and sh-PI3 groups, with five mice in each group. ES-2 cells stably expressing sh-NC or sh-PI3 were injected subcutaneously into the flank of each mouse. Tumor length and width were measured regularly using calipers, and tumor volume was estimated from the two perpendicular tumor dimensions. At the experimental endpoint, the mice were euthanized, and the tumors were excised, photographed, measured, and weighed. All animal procedures were approved by the Animal Ethics Committee of Tianjin Medical University Cancer Institute and Hospital.

### 2.16 Statistical analysis

All statistical tests were two-sided. Continuous variables are presented as the mean ± standard deviation or median, depending on their distribution. Comparisons between two groups were performed using an independent-samples t test or the Wilcoxon rank-sum test. Comparisons among three or more groups were conducted using one-way analysis of variance followed by multiple-comparison testing. Kaplan–Meier analysis and the log-rank test were used for survival comparisons. Cox regression was used to estimate hazard ratios and 95% confidence intervals. P values were adjusted when multiple testing was performed. P < 0.05 was considered statistically significant.

Data from the *in vitro* experiments are presented as the mean ± SEM. Comparisons among si-NC, siPI3-1, and siPI3–2 were performed using one-way ANOVA followed by Dunnett’s multiple-comparison test. Time-course data from the CCK-8 and xenograft growth experiments were analyzed using two-way ANOVA followed by multiple-comparison testing. Terminal tumor volume and tumor weight were compared between two groups using a two-sided unpaired Student’s t test. P < 0.05 was considered statistically significant.

## 3 Results

### 3.1 Construction of the ovarian cancer single-cell atlas and cell type annotation

We integrated scRNA-seq data from 12 ovarian tissue samples, comprising five age-matched nonmalignant ovarian tissue samples and tumor tissues from seven treatment-naïve patients with HGSOC. Following the stated quality-control procedure, 54,701 of the initial 65,820 cell barcodes were retained for downstream analysis, corresponding to an overall retention rate of 83.11%. A total of 2,000 highly variable genes were identified. The first 30 principal components were used for dimensionality reduction and clustering (**Figures 1A, B**). Before Harmony correction, cells from different samples showed partial separation. After correction, the cells were well mixed in the UMAP space, indicating a reduction in sample-related batch effects (**Figures 1C, D**). Unsupervised clustering identified 14 cell clusters (**Figure 1E**). Based on canonical marker genes, these clusters were assigned to eight major cell types: epithelial cells, T cells, NK cells, B cells, plasma cells, macrophages, endothelial cells, and fibroblasts (**Figure 1F**). Each cell type showed a distinct marker expression pattern. Epithelial cells expressed EPCAM, KRT19, and KRT8. T cells expressed CD3D, CD3E, and TRAC, while macrophages expressed LYZ, C1QA, and CD68. The expression patterns of the remaining markers also supported the cell type annotations (**Figure 1G**).

**Figure 1:**
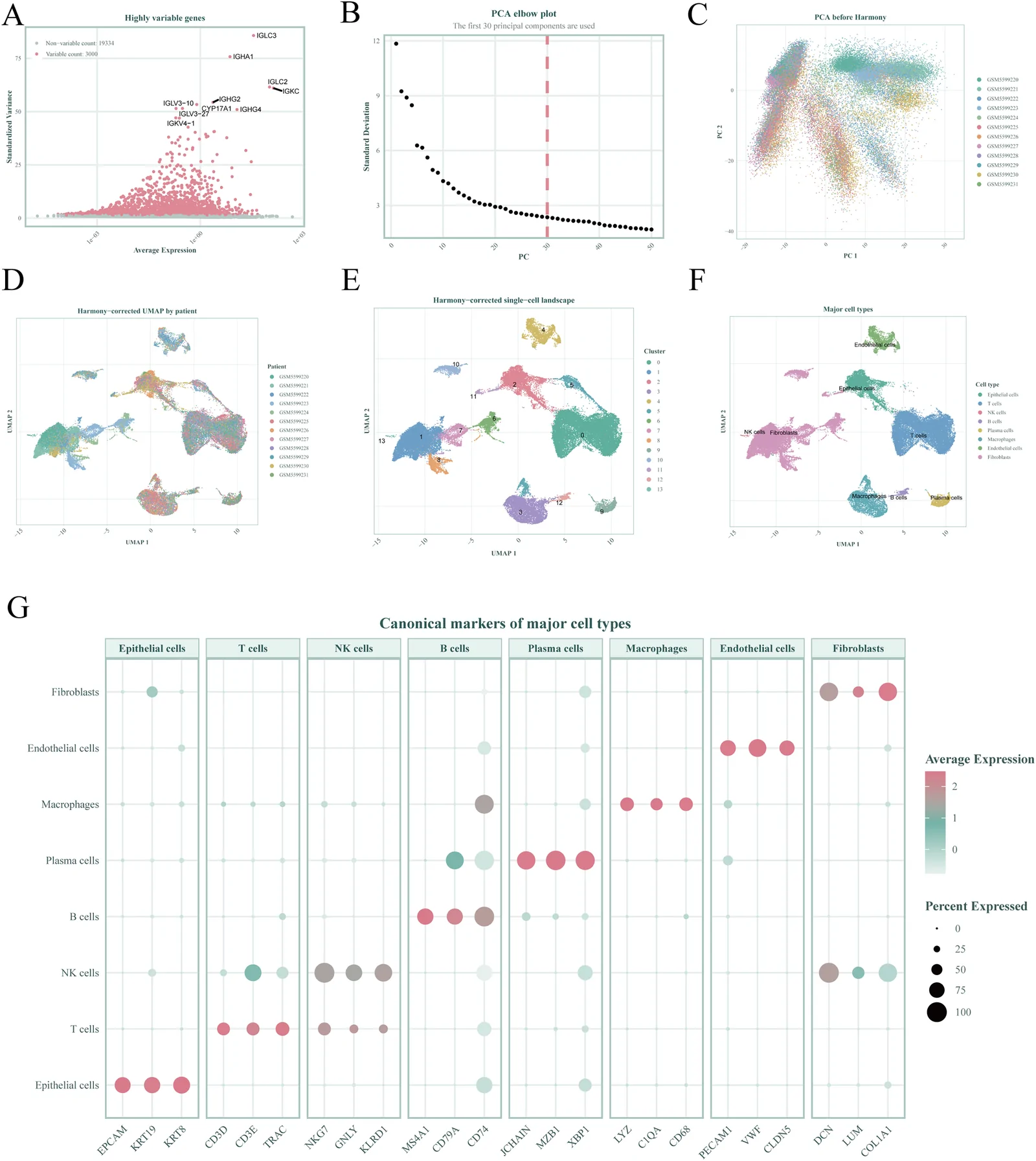
Construction of the ovarian cancer single-cell atlas and identification of major cell types. **(A)** Selection of highly variable genes. **(B)** Principal component analysis. **(C)** Cell distribution before Harmony correction. **(D)** UMAP distribution after Harmony correction. **(E)** Cell clusters identified by unsupervised clustering. **(F)** Annotation of major cell types. **(G)** Expression patterns of canonical marker genes across the identified cell types.

Cell composition varied across individual samples (**Supplementary Figure 1A**). HGSOC tissues contained higher proportions of epithelial cells and macrophages than nonmalignant ovarian tissues. In contrast, NK cells, endothelial cells, and fibroblasts were less abundant in HGSOC tissues. These differences were statistically significant. T-cell and B-cell proportions did not differ significantly between the two groups. Plasma cells showed an increasing trend in HGSOC tissues, although the difference did not reach statistical significance(**Supplementary Figure 1B**).

### 3.2 Macrophage heterogeneity and functional states in ovarian cancer

To characterize macrophage heterogeneity, macrophages were extracted and reclustered into eight subclusters (**Figure 2A**). Based on their transcriptional profiles and representative markers, these cells were subsequently assigned to four macrophage states: Resident Macro, Inflammatory Macro, SPP1+ TAM, and Activated Macro (**Figure 2B**). Their distributions differed between nonmalignant ovarian and HGSOC tissues (**Figure 2C**). Resident Macro was characterized by C1QC, FOLR2, and MRC1 expression, whereas Inflammatory Macro preferentially expressed FCN1, S100A9, and IL1B. SPP1+ TAM showed elevated expression of SPP1, GPNMB, and APOC1. Activated Macro was annotated at a higher functional level and included macrophages exhibiting proliferative and cell-cycle activity, represented by MKI67, together with interferon-responsive features represented by CXCL10 and ISG15 (**Figure 2D**). These genes reflected partially overlapping activation programs within this broad macrophage state and were not treated as a conjunctive marker combination that had to be expressed by every cell. The relative abundance of these macrophage states also differed between the two tissue groups. Inflammatory Macro was significantly less abundant in HGSOC tissues than in nonmalignant ovarian tissues (P = 0.006), whereas Activated Macro was significantly enriched in HGSOC tissues (P = 0.009). Resident Macro and SPP1+ TAM also tended to be more abundant in HGSOC tissues, although these differences did not reach statistical significance (both P = 0.104; **Figure 2E**). Functional enrichment analysis further demonstrated distinct biological programs among the four macrophage states. Activated Macro was associated with cell-cycle progression, chromosome segregation, DNA replication, E2F targets, and the G2M checkpoint. The predominance of these cell-cycle-related pathways indicated that proliferation was the principal functional program of Activated Macro, whereas the expression of CXCL10 and ISG15 reflected additional interferon-responsive heterogeneity within this broadly defined state. Accordingly, Activated Macro should be interpreted as an operational macrophage state containing related but not completely identical activation programs, rather than as a transcriptionally homogeneous terminal subtype. Inflammatory Macro was linked to inflammatory responses, responses to bacterial products, TNF-α signaling via NF-κB, and IL-17 signaling. SPP1+ TAM was primarily associated with phagosome, lysosome, cholesterol metabolism, and immune-regulatory processes, whereas Resident Macro was enriched in antigen processing, antigen presentation, and MHC-related functions (**Figure 2F**). These transcriptional, compositional, and functional differences highlight the heterogeneity of macrophage states within the ovarian cancer microenvironment.

**Figure 2:**
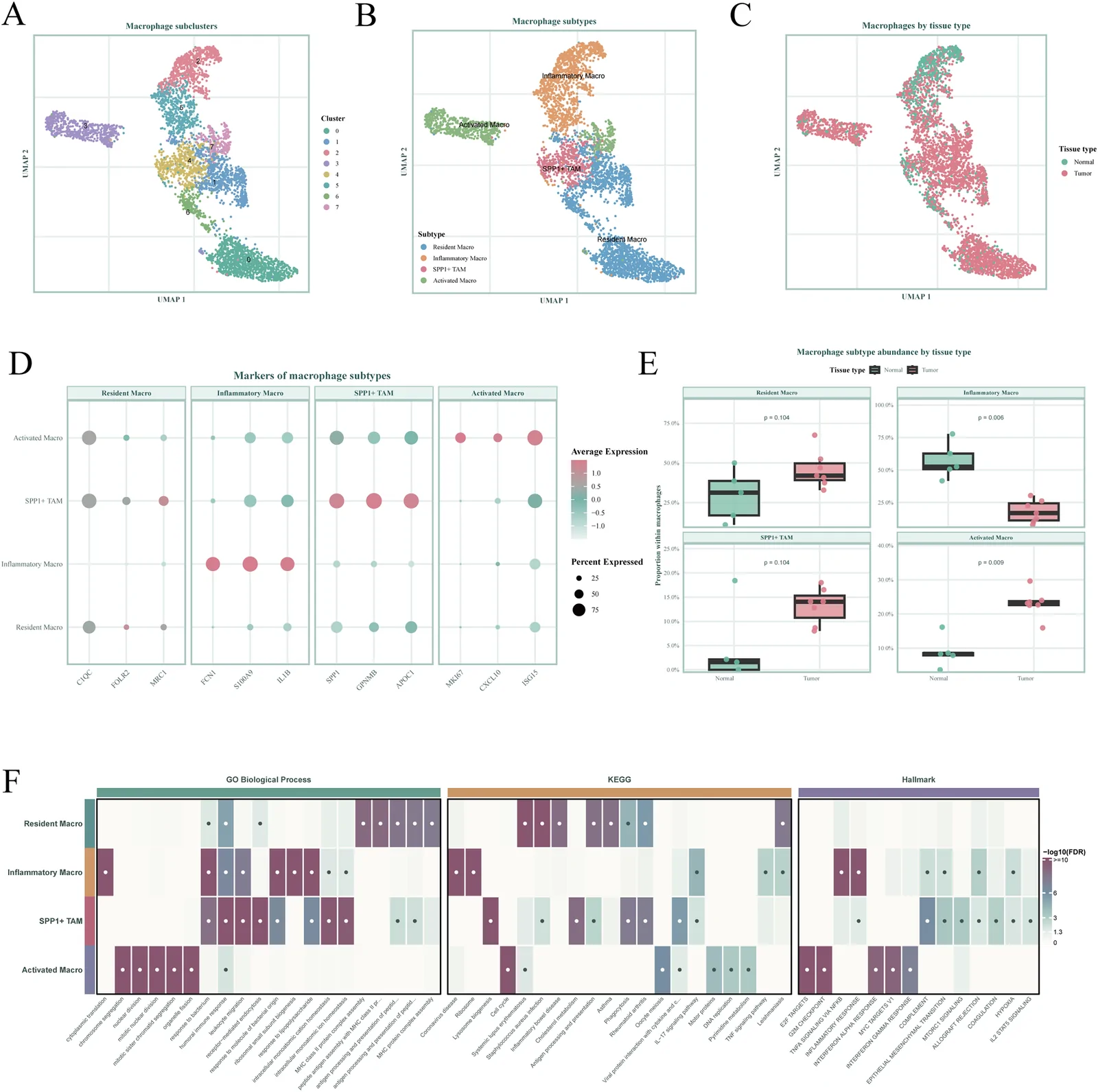
Identification and functional characterization of macrophage subtypes in ovarian cancer. **(A)** Secondary clustering of macrophages into eight subclusters. **(B)** UMAP visualization of the four annotated macrophage subtypes. **(C)** UMAP distribution of macrophages according to tissue type. **(D)** Dot plot showing the expression of representative marker genes across the four macrophage subtypes. Dot color represents average expression, and dot size indicates the percentage of cells expressing each gene. **(E)** Comparison of the relative abundance of each macrophage subtype between nonmalignant ovarian and HGSOC tissues. **(F)** GO biological process, KEGG, and Hallmark enrichment analyses of the four macrophage subtypes.

### 3.3 Identification of malignant epithelial cells by inferCNV

We used inferCNV to assess large-scale copy number alterations in epithelial cells. Compared with the reference cells, a subset of epithelial cells showed clear chromosomal gains and losses (**Supplementary Figure 2A**). The epithelial cells were then divided into five clusters according to their CNV profiles (**Supplementary Figure 2B**). Cluster 4 had the lowest CNV score and remained close to the reference level. The other four clusters showed higher CNV scores (**Supplementary Figure 2C**). Cluster 4 was therefore classified as normal epithelial cells, whereas Clusters 1, 2, 3, and 5 were defined as malignant epithelial cells.

### 3.4 Remodeling of cell–cell communication in ovarian cancer

CellChat was used to compare intercellular communication between nonmalignant ovarian tissues and HGSOC tissues. The final CellChat analysis included 24,299 cells from nonmalignant ovarian tissues and 26,973 cells from HGSOC tissues. Although the total numbers of cells were relatively comparable between the two groups, their cellular composition differed substantially. Fibroblasts were the predominant population in the nonmalignant group, accounting for 66.24% of cells (16,095/24,299), whereas T cells constituted the largest population in the HGSOC group, accounting for 48.87% (13,182/26,973). Tumor cells accounted for 15.05% of the HGSOC CellChat object (4,059/26,973). The complete numbers and relative proportions of all cell populations are provided in **Supplementary Table 1**. Most altered interactions were stronger in HGSOC tissues. Clear changes were observed among fibroblasts, macrophage subtypes, tumor cells, and T cells (**Figures 3A, B**). The interaction heatmaps also showed marked variation in both communication number and strength across cell populations (**Figure 3C**). In normal tissues, fibroblasts showed strong outgoing signals, while SPP1+ TAM received relatively high levels of incoming signals. Fibroblast-derived signaling became stronger in tumor tissues. SPP1+ TAM remained a major signal-receiving population. Activated Macro and Inflammatory Macro also showed increased incoming and outgoing activity (**Figure 3D**). At the pathway level, signaling was more broadly distributed in tumor tissues. MHC-I, COLLAGEN, MIF, APP, LAMININ, GALECTIN, CCL, and SPP1 were active across several cell populations. Fibroblasts and macrophage subtypes differed in their outgoing signaling programs. SPP1+ TAM and other macrophage populations received strong signals from surrounding cells (**Figures 3E, F**). Together, these findings indicate that communication between immune and stromal cells is extensively altered in ovarian cancer. Because the abundance and relative proportions of several cell populations differed between the two tissue groups, the comparative CellChat results were interpreted as network-level patterns under the observed cellular composition. Although population-size weighting was disabled, unequal cell abundance may still influence the detectability and stability of inferred interactions; therefore, differences in communication number and strength should not be attributed solely to changes in intrinsic cellular communication activity.

**Figure 3:**
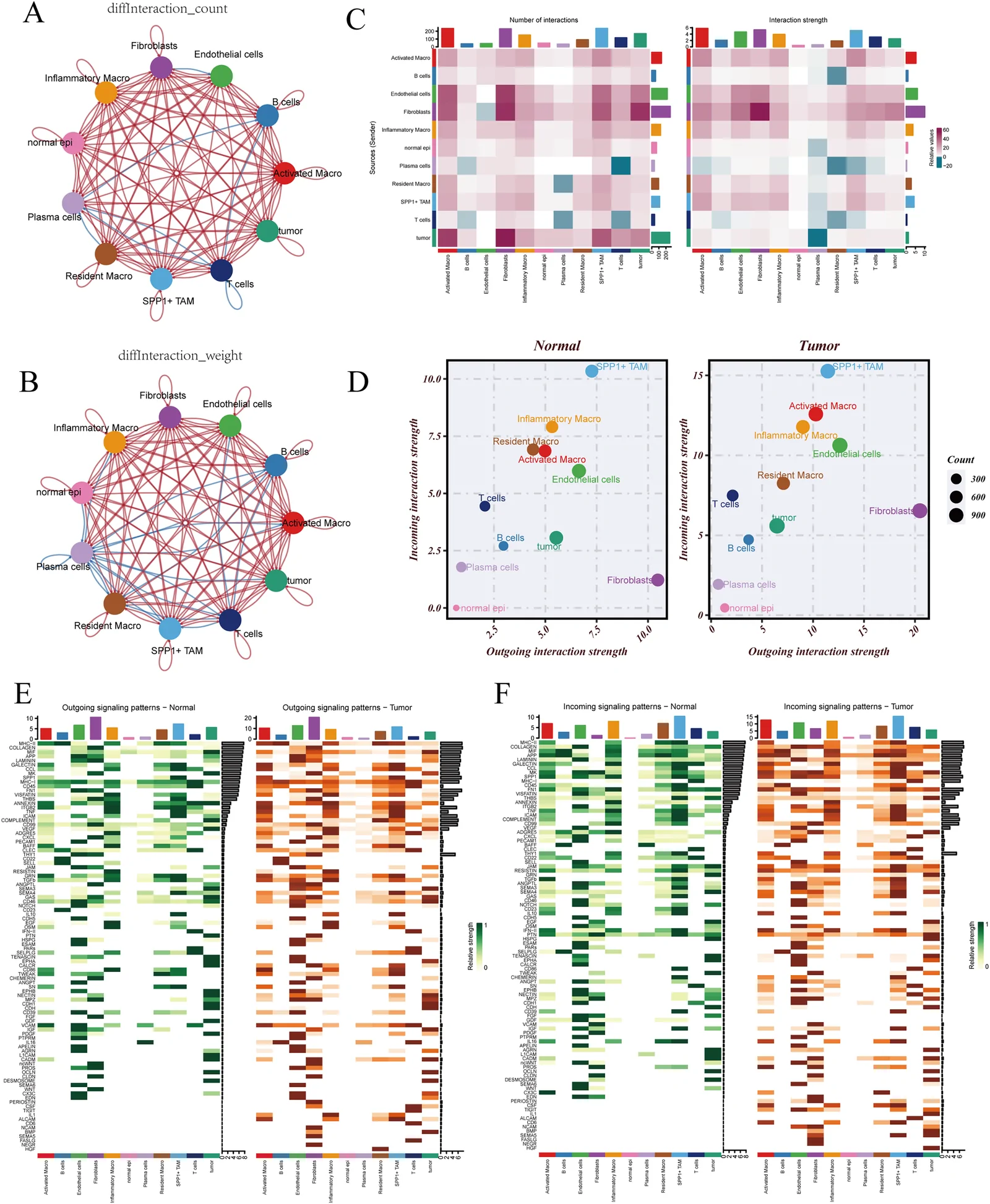
Differences in cell–cell communication networks between normal and ovarian tumor tissues. **(A, B)** Changes in the number and strength of cell–cell interactions between the two groups. Red indicates increased communication in tumors, whereas blue indicates decreased communication. **(C)** Number and strength of interactions among the identified cell types. **(D)** Incoming and outgoing signaling strength of each cell type in nonmalignant ovarian tissues and HGSOC tissues. **(E, F)** Incoming and outgoing signaling patterns across cell types in the two groups.

### 3.5 Macrophages engage in extensive signaling within the ovarian cancer microenvironment

Both nonmalignant ovarian tissues and HGSOC tissues contained complex communication networks. However, several interactions changed in number or strength in tumor tissues. T cells, tumor cells, fibroblasts, and macrophage subtypes were involved in many of these communication events (**Figure 4A**). We next examined the signals received by macrophages in tumor tissues. Several cell populations communicated with macrophage subtypes through SPP1-, MIF-, COLLAGEN-, FN1-, and HLA-related ligand–receptor pairs. SPP1+ TAM and the other macrophage subtypes received signals from a broad range of cells. Prominent interactions included SPP1 with integrins or CD44, MIF with CD74-associated receptors, and collagen with CD44 (**Figure 4B**). Macrophages also acted as important signal senders. All four macrophage subtypes communicated with immune, stromal, and tumor cells. Common signals involved SPP1, MIF, FN1, TNF, TGF-β, and chemokine-related pathways. Their outgoing patterns were not identical, suggesting that these macrophage populations may have different roles in the tumor microenvironment (**Figure 4C**).

**Figure 4:**
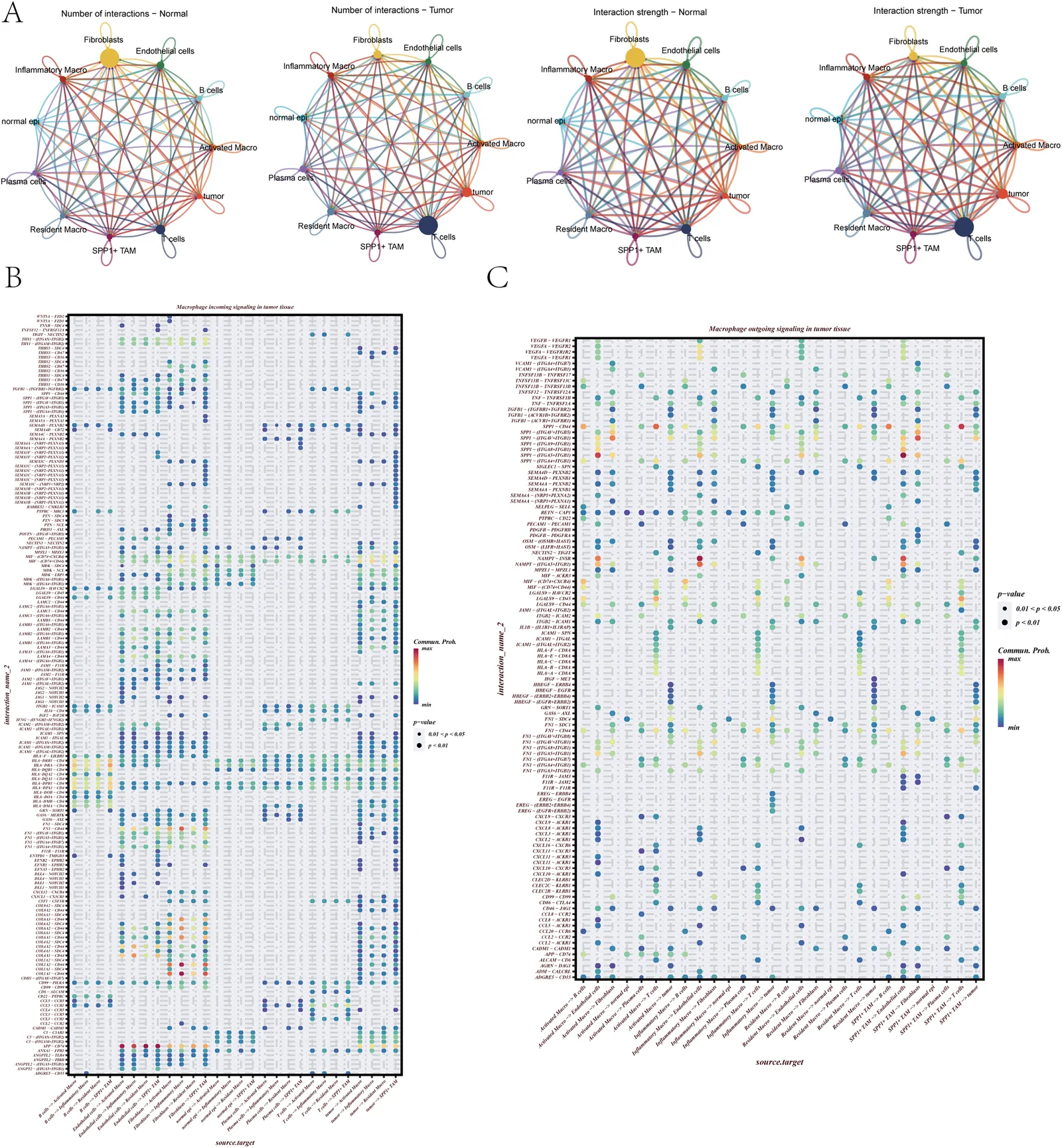
Macrophage-associated cell communication patterns in ovarian cancer. **(A)** Number and strength of intercellular interactions in nonmalignant ovarian tissues and HGSOC tissues. **(B)** Ligand–receptor signals transmitted from other cell types to macrophage subtypes in tumor tissues. **(C)** Ligand–receptor signals sent by macrophage subtypes to other cell types in tumor tissues. Bubble color indicates communication probability, and bubble size reflects statistical significance.

### 3.6 Altered macrophage signaling and reorganization of the SPP1 communication network

Macrophage-associated ligand–receptor signaling differed widely between nonmalignant ovarian tissues and HGSOC tissues. More interactions were increased than decreased in tumors, and these changes involved several recipient cell populations. Signals related to SPP1, MIF, FN1, HLA molecules, and chemokines were particularly evident (**Figure 5A**). SPP1 interactions with CD44 or integrin receptors were detected across several macrophage subtypes. By contrast, some interactions involving collagen, APP, and chemokines were weaker in tumor tissues (**Figure 5B**). Because SPP1 signaling was prominent in the differential network, we examined its communication structure in greater detail. In normal tissues, SPP1+ TAM already occupied a central position and communicated with several cell types (**Figures 5E, F**). The network showed a broader pattern in tumor tissues. Tumor cells, endothelial cells, fibroblasts, and several macrophage subtypes were involved (**Figures 5C, D**). SPP1+ TAM remained a major signaling node, but its connections with surrounding cells became more complex. These findings suggest that SPP1-mediated communication may contribute to remodeling of the ovarian cancer microenvironment.

**Figure 5:**
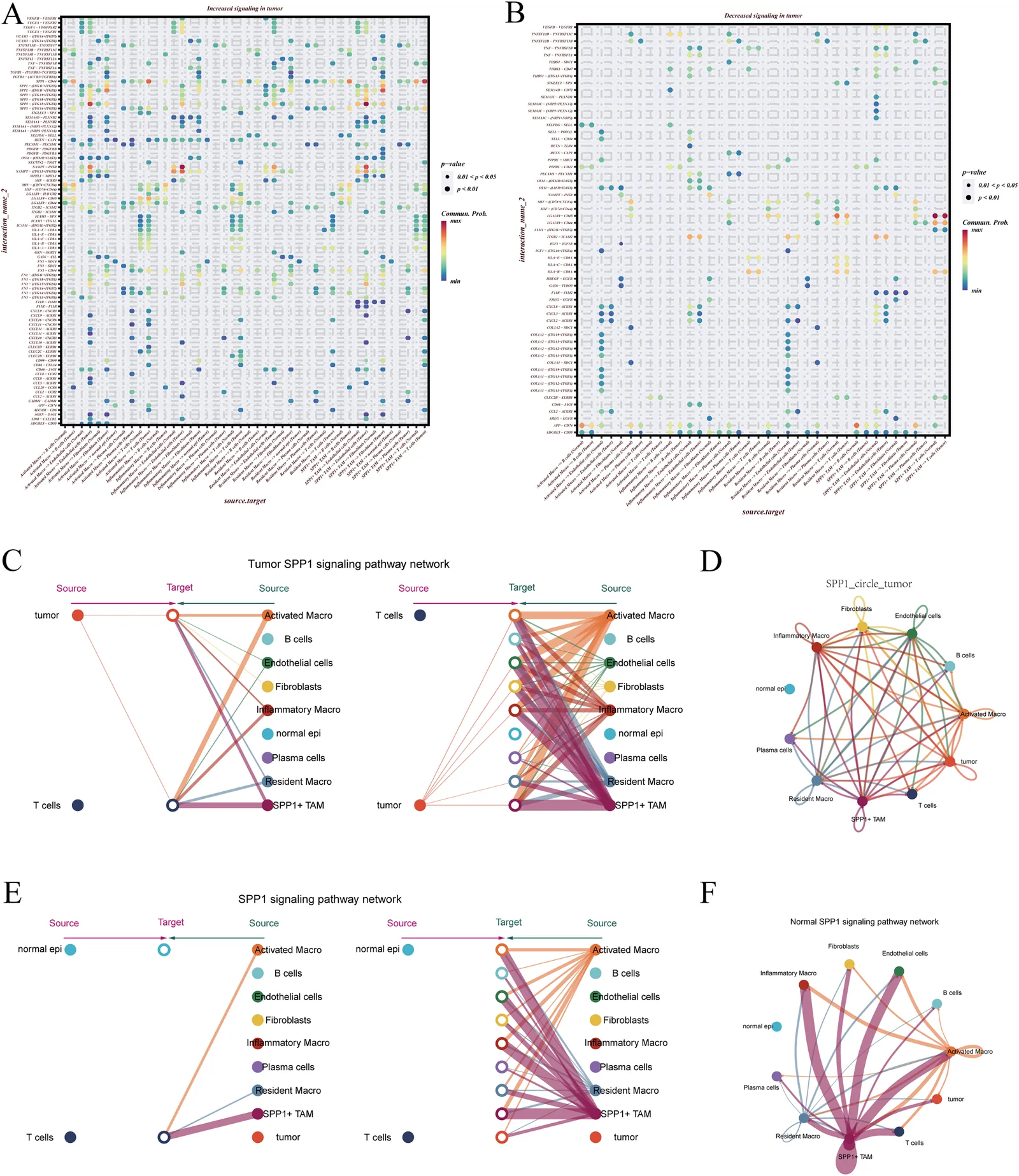
Changes in macrophage-associated ligand–receptor signaling and the SPP1 communication network in ovarian cancer. **(A, B)** Macrophage-associated ligand–receptor interactions that were increased or decreased in HGSOC tissues relative to nonmalignant ovarian tissues. Bubble color indicates the inferred communication probability, and bubble size represents statistical significance. **(C, D)** Hierarchical and circular representations of the SPP1 signaling network in HGSOC tissues, respectively. **(E, F)** Hierarchical and circular representations of the SPP1 signaling network in nonmalignant ovarian tissues, respectively. In Panels **(C–F)**, nodes represent individual cell populations, and node colors identify the corresponding cell types. Arrows indicate the direction of signal transmission from sender to receiver populations. Edge colors correspond to the source cell populations, whereas edge width is proportional to the inferred SPP1 communication strength. In the hierarchical plots, filled and open nodes denote signal-sending and signal-receiving cell populations, respectively.

### 3.7 SPP1 signaling-related genes define two molecular subtypes of ovarian cancer

Using the fixed 11-gene set derived from the SPP1 communication network identified by CellChat, consensus clustering was performed in the TCGA-OV cohort. The CDF curves and changes in the area under the curves indicated that the clustering result was relatively stable at K = 2 (**Figures 6A, B**). The consensus matrix also showed clear within-group consistency. Patients were therefore classified into SPP1-Low and SPP1-High subtypes (**Figure 6C**). SPP1 pathway activity differed between the two groups. The SPP1-High subtype had a higher pathway score (P = 0.0154; **Figure 6D**). The heatmap also showed distinct expression patterns of SPP1, CD44, and several integrin receptor genes. These included ITGB6, ITGB5, ITGB1, ITGAV, ITGA4, ITGA5, ITGA8, ITGA9, and ITGB3 (**Figure 6E**). Patients in the SPP1-High group had shorter overall survival. Their risk of death was higher than that of the SPP1-Low group (HR = 1.74, 95% CI: 1.35–2.23, P < 0.001; **Figure 6F**). PCA further showed partial separation between the two subtypes at the transcriptomic level (**Figure 6G**). These findings suggest that SPP1-based classification captures biological differences with potential prognostic relevance.

**Figure 6:**
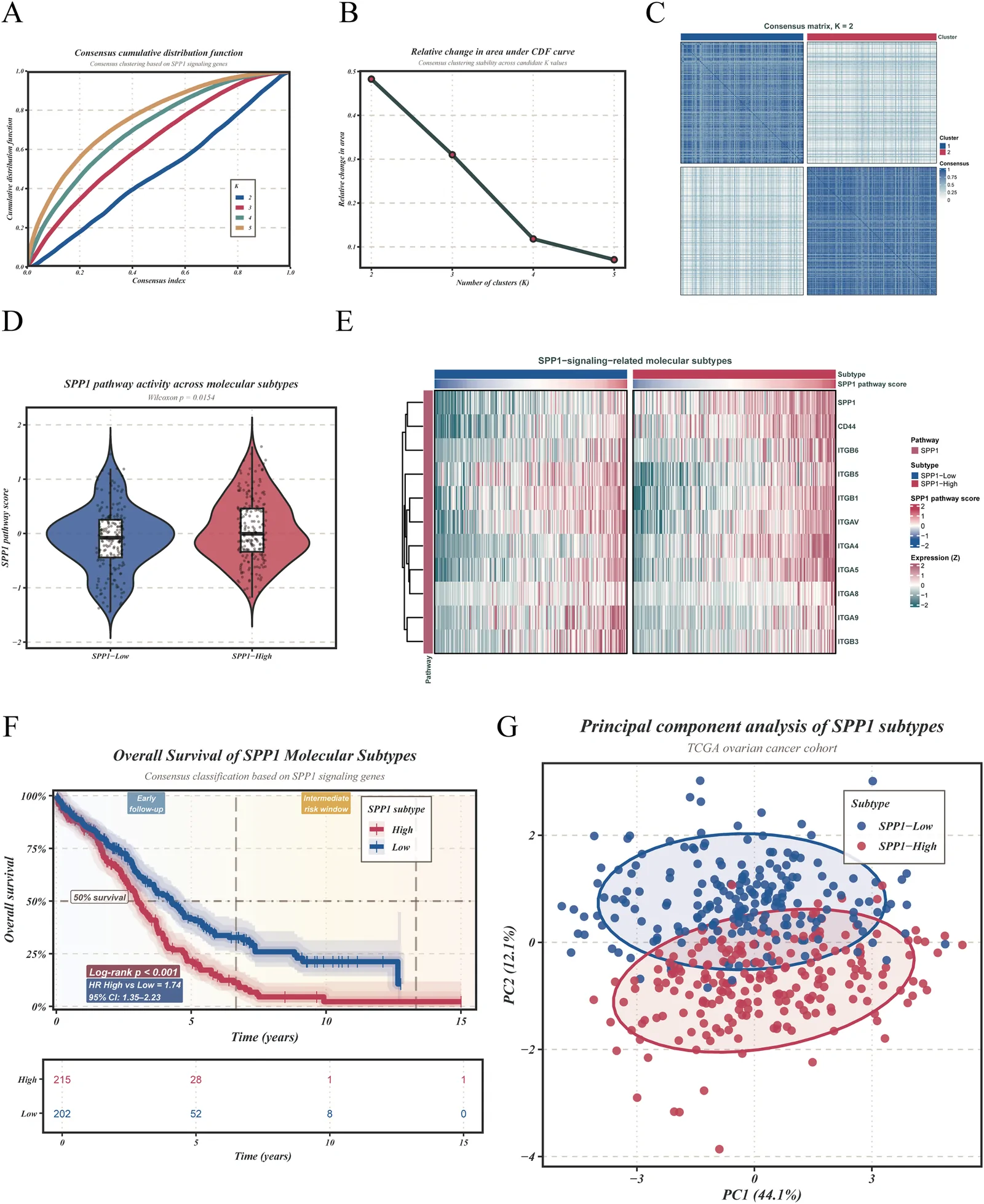
Molecular classification of ovarian cancer based on the CellChat-derived SPP1 ligand–receptor gene set. **(A)** Consensus cumulative distribution function curves for different cluster numbers. **(B)** Relative change in the area under the CDF curve. **(C)** Consensus clustering matrix at K = 2. **(D)** Comparison of SPP1 pathway activity scores between the two molecular subtypes. **(E)** Expression patterns of SPP1 and its receptor-related genes across the two subtypes. **(F)** Overall survival analysis of the SPP1-High and SPP1-Low subtypes. **(G)** Principal component analysis of the two subtypes.

### 3.8 SPP1 molecular subtypes differ in immune context and prognostic features

The two SPP1 subtypes showed clear molecular and immune differences. Differential genes were mainly enriched in humoral immunity, neutrophil chemotaxis, phagosome, complement and coagulation cascades, IL-17 signaling, and cytokine–receptor interactions (**Figures 7A, B**). The top 60 genes also showed distinct expression patterns between the two groups (**Figure 7C**). Cox analysis identified PI3, VSIG4, ALOX5AP, RARRES1, and SAA1 as risk-related genes, whereas IGKC, IGLC3, CXCL9, and several T-cell receptor genes were associated with better survival (**Figure 7D**). The SPP1-High subtype showed stronger immune-related activity, including T-cell receptor, MHC class I, immunoglobulin, and cytokine signaling. The SPP1-Low subtype was more closely linked to cell cycle, mismatch repair, ciliary movement, and metabolism (**Figures 7E, F**). CIBERSORT further showed higher levels of memory B cells, M2 macrophages, and resting dendritic cells in the SPP1-High group, but lower levels of naïve B cells and plasma cells (**Supplementary Figure 3**). These findings indicate that the two subtypes differ in prognosis, pathway activity, and immune composition.

**Figure 7:**
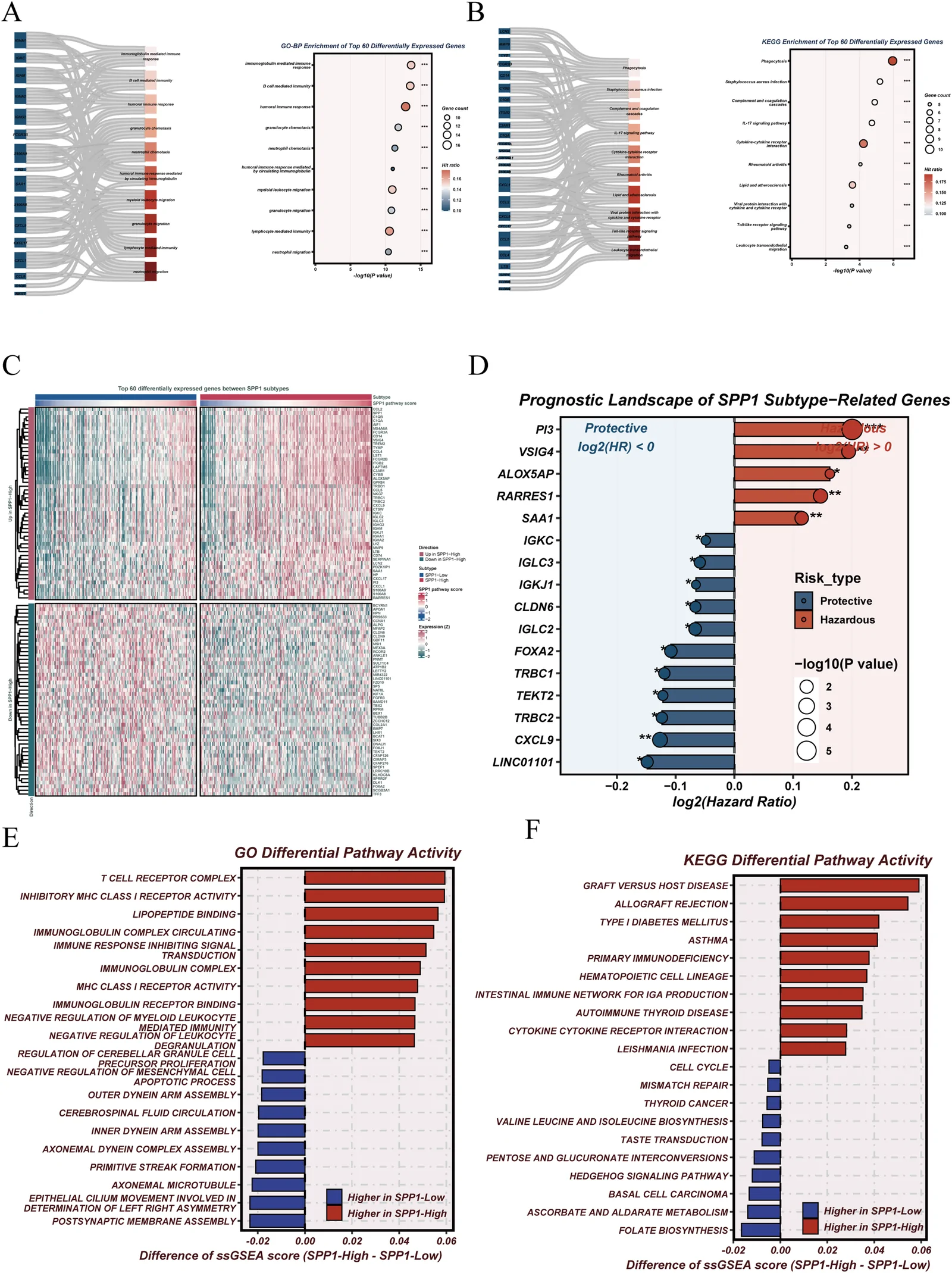
Transcriptional features, functional differences, and prognostic genes associated with the SPP1 molecular subtypes. **(A, B)** GO biological process and KEGG enrichment analyses of differentially expressed genes between the two SPP1 subtypes. **(C)** Heatmap showing the top 60 differentially expressed genes between the two subtypes. **(D)** Univariate Cox regression analysis of SPP1 subtype-related genes. **(E, F)** Differences in GO and KEGG pathway activities between the two subtypes. Red indicates higher activity in the SPP1-High subtype, whereas blue indicates higher activity in the SPP1-Low subtype.

### 3.9 Construction and validation of an SPP1 subtype-related prognostic model

We used LASSO–Cox, GBM–Cox, random survival forest, and ABESS–Cox to screen candidate genes. LASSO–Cox retained 12 genes. VSIG4 and PI3 had relatively large positive coefficients, whereas CXCL9, LINC01101, and TRBC2 had negative coefficients (**Figures 8A–C**). The GBM–Cox model reached a low cross-validation error at approximately 301 trees. PI3, VSIG4, and CXCL9 ranked among the most important variables (**Figures 8D, E**). The out-of-bag error of the random survival forest gradually stabilized as the number of trees increased. PI3 showed the highest importance, followed by VSIG4 and TEKT2 (**Figures 8F, G**). ABESS–Cox retained a smaller set of features (**Figures 8H, I**). Among the four models, GBM–Cox achieved the highest C-index of 0.768. The C-index values of LASSO–Cox, ABESS–Cox, and random survival forest were 0.660, 0.652, and 0.630, respectively (**Figure 8J**). Bootstrap resampling yielded similar results (**Figure 8K**). GBM–Cox was therefore selected as the final prognostic model. Several genes, including PI3, VSIG4, TRBC1, TEKT2, and RARRES1, were retained by more than one algorithm (**Figure 8L**). The model was then evaluated in TCGA-OV and three independent cohorts. High-risk patients had shorter overall survival in all four datasets. The hazard ratios were 1.85 in TCGA-OV, 1.94 in GSE140082, 1.54 in GSE26712, and 1.41 in GSE13876. All differences were statistically significant (**Figure 9A**). Time-dependent ROC analysis further assessed model performance. In TCGA-OV, the 1-, 3-, and 5-year AUC values were 0.636, 0.665, and 0.660, respectively. In GSE140082, the 1- and 3-year AUC values were 0.748 and 0.723. The AUC values were lower in GSE26712 and GSE13876, but the model still retained some ability to distinguish patient risk (**Figure 9B**). PCA showed varying degrees of separation between the high- and low-risk groups across the four cohorts (**Figure 9C**). These results support the prognostic value of the GBM–Cox model in ovarian cancer.

**Figure 8:**
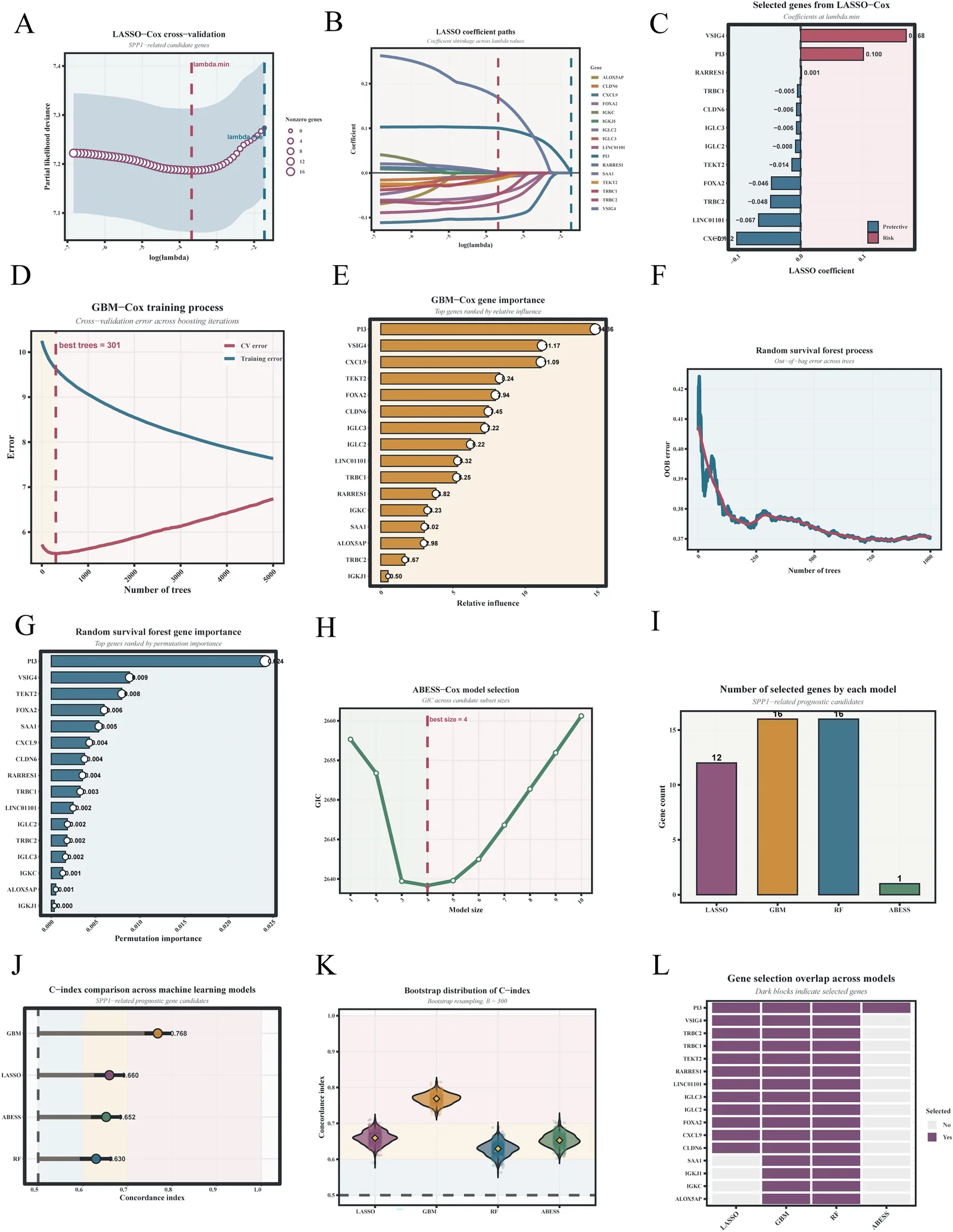
Machine learning-based selection of prognostic genes associated with the SPP1 subtypes. **(A)** Cross-validation curve of the LASSO–Cox model. **(B)** LASSO coefficient profiles. **(C)** Genes selected by LASSO–Cox and their regression coefficients. **(D)** Training process of the GBM–Cox model. **(E)** Gene importance ranking in the GBM–Cox model. **(F)** Out-of-bag error of the random survival forest model. **(G)** Gene importance ranking in the random survival forest model. **(H)** Selection of the optimal feature number in the ABESS–Cox model. **(I)** Number of genes selected by each model. **(J)** Comparison of C-index values across the four models. **(K)** Distribution of C-index values obtained by bootstrap resampling. **(L)** Overlap of genes selected by different models.

**Figure 9:**
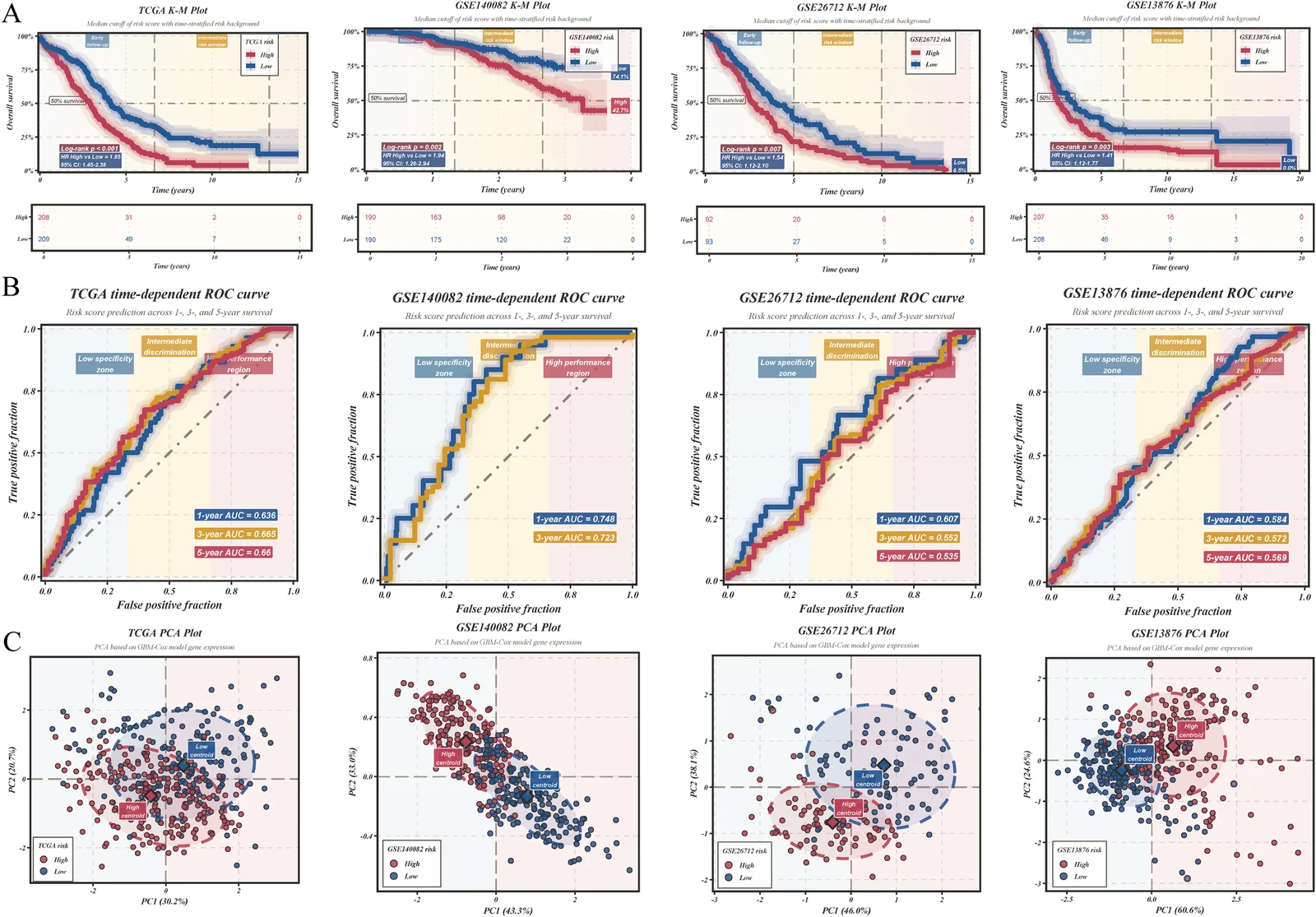
Prognostic performance of the GBM–Cox model in the training and external validation cohorts. **(A)** Kaplan–Meier survival curves for the high- and low-risk groups in the TCGA-OV, GSE140082, GSE26712, and GSE13876 cohorts. **(B)** Time-dependent ROC curves for 1-, 3-, and 5-year overall survival in each cohort. Because of the limited follow-up time in GSE140082, only the 1- and 3-year ROC curves were evaluated. **(C)** Principal component analysis based on the expression of model genes.

### 3.10 PI3 knockdown in tumor cells promotes an M1-like phenotype in cocultured macrophages

qRT-PCR confirmed that both siRNAs reduced PI3 expression in ES-2 and SKOV3 cells (**Figure 10A**). We then established an indirect coculture system using tumor cells and THP-1-derived M0 macrophages. The experiment included M0, si-NC, and si-PI3 groups. Macrophages and culture supernatants were collected after 48 h of coculture (**Figure 10B**). Compared with the si-NC group, macrophages cocultured with PI3-silenced tumor cells showed higher expression of TNF, IL1B, CXCL10, and CD86 (**Figure 10C**). In contrast, CD163, MRC1, CCL18, and IL10 were reduced (**Figure 10D**). Similar changes were observed in both the ES-2 and SKOV3 coculture systems. The ELISA results followed the same pattern. TNF-α and IL-12p70 levels were increased in the si-PI3 group, whereas IL-10 and CCL18 levels were decreased (**Figures 10E, F**). These findings indicate that PI3 knockdown in ovarian cancer cells alters their effect on macrophages and favors an M1-like transcriptional and secretory phenotype.

**Figure 10:**
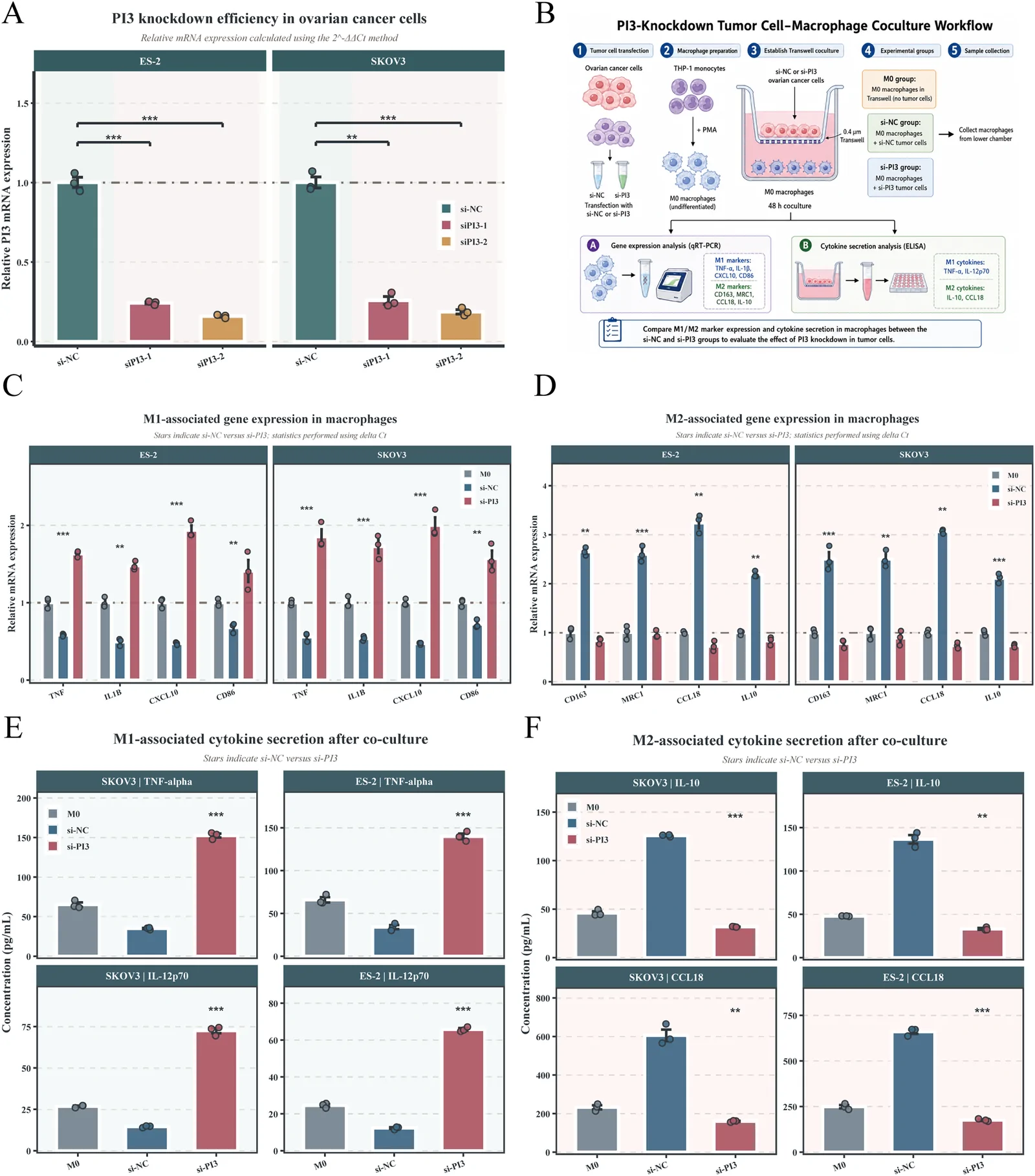
Effects of PI3 knockdown in tumor cells on the phenotype of cocultured macrophages. **(A)** PI3 knockdown efficiency in ES-2 and SKOV3 cells measured by qRT-PCR. **(B)** Transwell coculture workflow using PI3-silenced tumor cells and THP-1-derived M0 macrophages. **(C, D)** Expression of M1- and M2-related genes in macrophages after coculture. **(E, F)** Levels of M1- and M2-related cytokines in the culture supernatants measured by ELISA. Asterisks indicate differences between the si-NC and si-PI3 groups. **P < 0.01 and ***P < 0.001. Data are presented as the mean ± SEM from three independent biological experiments. Comparisons among the three groups were performed using one-way ANOVA followed by Dunnett’s multiple-comparison test, with si-NC as the reference group.

### 3.11 PI3 silencing suppresses SPP1 expression and ovarian cancer progression *in vitro* and *in vivo*

To further investigate the functional role of PI3 in ovarian cancer, PI3 was silenced using two independent siRNAs in SKOV3 and ES-2 cells. SPP1 mRNA expression was significantly decreased following PI3 knockdown in both cell lines (**Figure 11A**). Consistently, ELISA of tumor-cell conditioned medium showed that secreted SPP1 levels were markedly reduced in the siPI3–1 and siPI3–2 groups after normalization to viable cell number (**Figure 11B**). These results establish an experimental association between PI3 expression and tumor-cell-derived SPP1.

**Figure 11:**
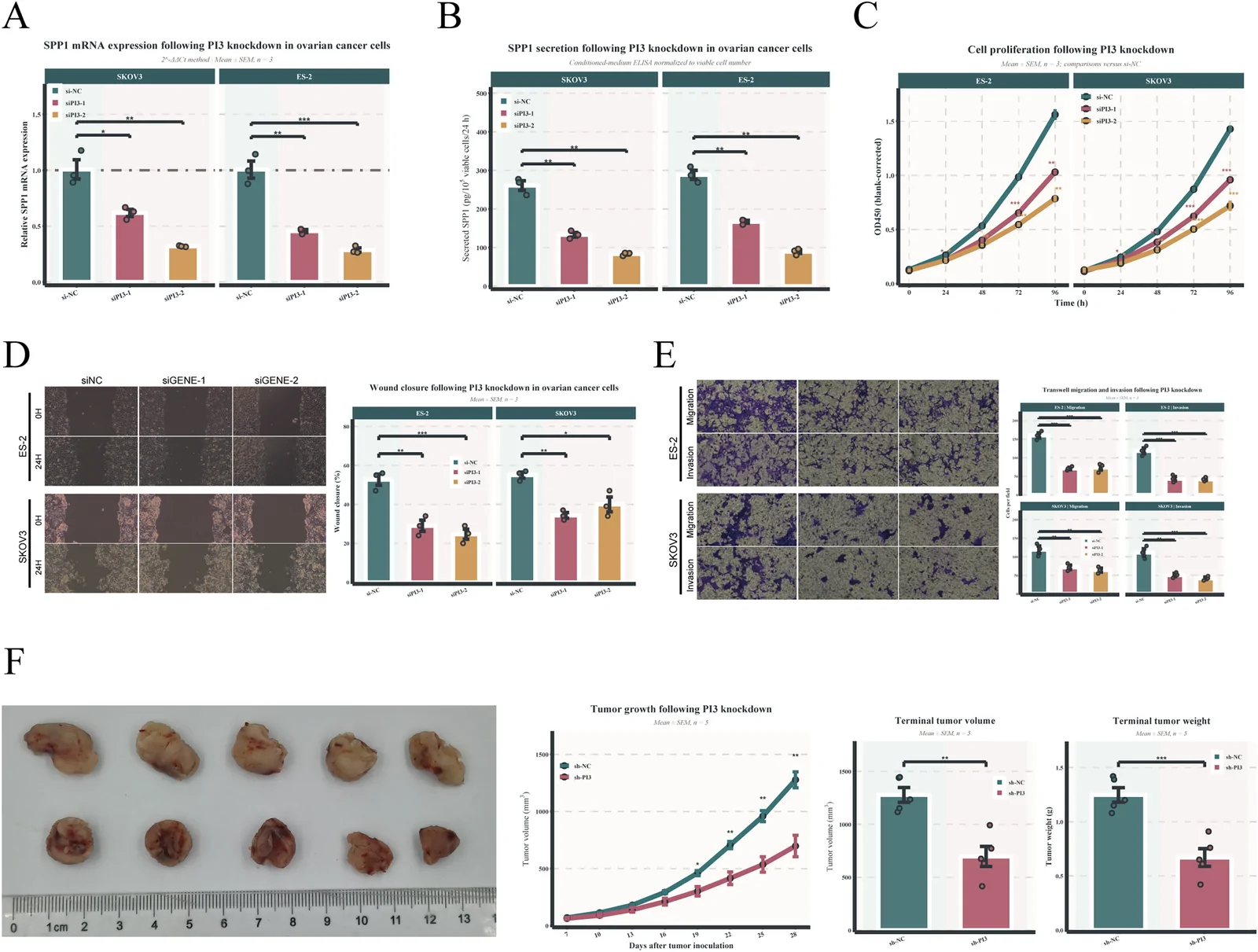
PI3 knockdown suppresses SPP1 expression and the malignant phenotypes of ovarian cancer cells. **(A)** Relative SPP1 mRNA expression in SKOV3 and ES-2 cells following transfection with si-NC, siPI3-1, or siPI3-2. **(B)** Secreted SPP1 levels in conditioned medium collected from PI3-silenced ovarian cancer cells, as determined by ELISA and normalized to viable cell number. **(C)** Proliferation of ES-2 and SKOV3 cells measured using CCK-8 assays. **(D)** Representative images and quantitative analysis of wound-healing assays following PI3 knockdown. **(E)** Representative images and quantitative analysis of Transwell migration and invasion assays. **(F)** Representative xenograft tumors, tumor growth curves, terminal tumor volumes, and terminal tumor weights in the sh-NC and sh-PI3 groups. Data are presented as the mean ± SEM. The *in vitro* experiments included three independent biological replicates, and the animal experiment included five mice per group. *P < 0.05, **P < 0.01, and ***P < 0.001. Comparisons among si-NC, siPI3-1, and siPI3–2 were performed using one-way ANOVA followed by Dunnett’s multiple-comparison test, with si-NC as the reference group.

CCK-8 assays showed that both PI3-targeting siRNAs progressively inhibited the proliferation of ES-2 and SKOV3 cells (**Figure 11C**). Wound-healing assays further demonstrated that PI3 knockdown significantly reduced the wound-closure rate in both cell lines (**Figure 11D**). Similarly, the numbers of migrating and invading cells were markedly decreased after PI3 silencing, indicating impaired migratory and invasive capacities (**Figure 11E**). The effect of PI3 knockdown was further examined in an ES-2 subcutaneous xenograft model. Tumors derived from sh-PI3 cells grew more slowly than those derived from sh-NC cells. At the experimental endpoint, the sh-PI3 group also showed significantly lower tumor volume and tumor weight (**Figure 11F**). Together, these findings support a tumor-promoting role of PI3 in ovarian cancer and provide a direct experimental link between PI3 and tumor-cell-derived SPP1.

## 4 Discussion

The ovarian cancer microenvironment is not simply a collection of different cell types. It is a tissue state maintained by continuous signaling among immune, malignant epithelial, and stromal cells. Secreted factors, extracellular matrix components, and ligand–receptor interactions connect local inflammation and tissue remodeling with tumor progression (21). Cell abundance or the expression of a single gene may therefore provide only a partial view of microenvironmental behavior. Communication analysis adds another layer by distinguishing where a signal originates and which cells are likely to receive it (22).

Macrophages are important participants in this regulatory network. The conventional M1/M2 framework describes two opposing functional tendencies, but macrophages in tumors are exposed simultaneously to hypoxia, metabolic competition, inflammatory mediators, and tumor-derived factors. Their phenotypes are therefore better represented as a continuum rather than two fixed states (23, 24). Tissue-resident, inflammatory, interferon-responsive, proliferative, and lipid-related programs may coexist within the same tumor. Macrophage subtypes should thus be viewed as context-dependent functional tendencies rather than completely separate terminal populations (25). Within this framework, Activated Macro was used as a broad operational annotation encompassing a predominantly proliferative and cell-cycle-active macrophage population with additional interferon-responsive features. The designation should not be interpreted as indicating that MKI67, CXCL10, and ISG15 represent the same molecular program or that all cells within this state are transcriptionally homogeneous. Although further subdivision may reveal additional heterogeneity, separating these cells solely according to a small number of individual markers could over-discretize a continuous activation spectrum and generate small subpopulations with limited stability. This heterogeneity also suggests that individual macrophage populations may influence tumor behavior through different ligand-receptor combinations.

SPP1 provides a useful entry point for understanding this form of multicellular regulation. It acts as both a secreted signaling molecule and an extracellular matrix-associated protein. Through CD44 and several integrins, SPP1 can affect adhesion, migration, survival, and inflammatory responses (26, 27). Because these receptors are present on immune cells, tumor cells, fibroblasts, and endothelial cells, the activity of SPP1 is unlikely to be restricted to one cell population. It may be more appropriate to regard SPP1 as part of a multicellular communication module linking immune regulation with tissue remodeling rather than merely as a marker of one macrophage subset.

SPP1-expressing macrophages have been described in several malignancies and have been linked to matrix deposition, angiogenesis, immune exclusion, and treatment resistance (28, 29). Their effects, however, may depend on the receptor repertoire and cellular context. SPP1 binding to CD44 may be more closely related to migration, survival, and immune regulation, whereas different integrin combinations may connect SPP1 to focal adhesion, cytoskeletal organization, and extracellular matrix signaling (30, 31). Similar levels of SPP1 expression may therefore produce different biological consequences across tissues. Evaluating ligand origin, receptor distribution, and signaling direction together is likely to be more informative than measuring SPP1 expression alone.

The association between SPP1-related molecular states and patient outcome may arise because this signal reflects the broader tissue ecosystem. In bulk transcriptomic data, SPP1-related expression is contributed by malignant epithelial cells, macrophages, and stromal populations rather than a single cellular source. A classification based on this signal may therefore capture immune activation, matrix remodeling, and tumor-cell adaptation at the same time. This provides a broader biological basis than a single-gene classifier, but the interpretation should remain cautious. Strong immune-related expression does not necessarily indicate effective antitumor immunity. It may instead reflect persistent inflammation, myeloid accumulation, or immune dysfunction (32, 33).

Machine learning can extract prognostic information from correlated candidate genes, but its value depends on biological interpretability. Algorithms differ in their handling of nonlinear effects, variable interactions, and redundant features, so different performance across models is not unexpected (34, 35). Selecting variables from an SPP1-related state gives the model a clearer microenvironmental context. Even so, a risk score should not be considered a substitute for established clinical factors. Prospective validation is still needed, together with an assessment of whether the model adds information beyond stage, residual disease, treatment, and other routine variables.

PI3 may represent another route through which tumor cells influence both their intrinsic malignant behavior and macrophage phenotype. PI3 encodes a secreted protease inhibitor involved in local inflammation, proteolytic balance, and epithelial barrier regulation, although its role appears to vary across tumor types (36, 37). In the present study, PI3 knockdown suppressed ovarian cancer cell proliferation, wound closure, migration, and invasion. Stable PI3 knockdown also delayed ES-2 xenograft growth and reduced terminal tumor volume and weight. These findings provide functional support for the prognostic importance of PI3 identified by multiple machine learning models and suggest that PI3 contributes to ovarian cancer progression.

Importantly, PI3 knockdown decreased both SPP1 mRNA expression and secreted SPP1 levels in ovarian cancer cells, thereby establishing an experimental link between PI3 and the SPP1-related communication network. Reduced tumor-cell-derived SPP1 may partly contribute to the impaired malignant phenotypes and the shift toward an inflammatory or M1-like state observed in cocultured macrophages. Nevertheless, the current findings do not demonstrate that PI3 directly regulates SPP1 transcription or that the macrophage response is mediated exclusively by SPP1. PI3 may also influence macrophage phenotype by altering extracellular protease activity, inflammatory mediators, or other tumor-derived soluble factors. Further rescue experiments involving PI3 re-expression or recombinant SPP1, together with SPP1 or receptor blockade, are required to clarify the causal relationship between PI3, SPP1, and macrophage regulation.

The macrophage changes observed *in vitro* should also not be equated with complete classical M1 polarization. THP-1-derived macrophages reproduce some inflammatory and immune-regulatory features, but they remain different from patient-derived tumor-associated macrophages (38, 39). In human tumors, macrophages are additionally shaped by fibroblasts, endothelial cells, hypoxia, lipids, and extracellular matrix components. It is therefore more accurate to describe the effect of PI3 knockdown as a shift toward an inflammatory or M1-like state rather than full conversion into classical M1 macrophages.

More broadly, translational biomedical research increasingly draws on complementary forms of evidence, including diagnostic-test evaluation, bioactive-compound screening, AI-assisted structural modeling, bibliometric and population-based cancer analyses, and mechanistic preclinical testing of candidate interventions (40–44). Recent oncology studies have further demonstrated how single-cell transcriptomics and machine-learning frameworks can connect cellular heterogeneity and immune-metabolic states with prognostic stratification, therapeutic response assessment, and, where appropriate, functional validation. Such integrative strategies have been applied to immunogenic cell death, epithelial heterogeneity, lactylation-related immune-metabolic dysregulation, and fibroblast subtypes in lung adenocarcinoma (45–48). Although these approaches address different diseases and biological questions, they collectively emphasize the value of aligning the analytical method with the research question and supporting computational or observational findings with independent evidence. This principle also informed our integration of single-cell analysis, prognostic modeling, and functional validation in the present study.

Several limitations should be considered. Cell communication was inferred mainly from transcript-level ligand and receptor expression and does not directly demonstrate protein binding, receptor activation, or downstream signal transmission. In addition, the cell-type composition was not balanced between nonmalignant ovarian and HGSOC tissues. Although communication probabilities were calculated without population-size weighting, differences in cell abundance may still affect the statistical power and stability of the inferred communication networks. Spatial proximity is also essential for effective communication, yet this information is lost in dissociation-based scRNA-seq data (49, 50). Spatial transcriptomics, multiplex immunofluorescence, or *in situ* protein detection could help confirm whether the predicted SPP1-related populations are physically adjacent. Although the newly added *in vitro* and xenograft experiments support a tumor-promoting role of PI3, its mechanistic relationship with SPP1 requires further validation using PI3 re-expression, recombinant SPP1 rescue, SPP1 or receptor blockade, and patient-derived macrophages. The prognostic model also requires validation in prospective cohorts with more complete clinical information.

Taken together, SPP1 appears to function as a cross-cellular communication node connecting macrophages, malignant epithelial cells, and stromal populations rather than simply as an expression marker of one cell subset. PI3 may participate in the paracrine regulation of myeloid-cell states by ovarian cancer cells. Defining the relevant SPP1 receptor combinations, PI3-associated secretory changes, and their spatial organization may help clarify how immune conditions and tumor phenotypes are maintained within the ovarian cancer microenvironment.

## Data Availability

The original contributions presented in the study are included in the article/**Supplementary Material**. Further inquiries can be directed to the corresponding authors.

## Supplementary material

Supplementary Figure 1 Differences in the composition of major cell types between nonmalignant ovarian tissues and HGSOC tissues. **(A)** Relative proportions of major cell types in each sample. **(B)** Comparison of cell-type proportions between the normal and tumor groups.

Supplementary Figure 2 Identification of malignant epithelial cells using inferCNV. **(A)** CNV landscape of epithelial cells, with normal cells used as the reference. **(B)** K-means clustering of epithelial cells based on inferred CNV profiles. **(C)** Distribution of CNV scores across the five clusters.

Supplementary Figure 3 Immune cell infiltration patterns of the SPP1 molecular subtypes. **(A)** Relative proportions of 22 immune cell types estimated by CIBERSORT in the SPP1-Low and SPP1-High subtypes. **(B)** Comparison of immune cell infiltration between the two subtypes. Only samples with a CIBERSORT P value below 0.05 were included. *P < 0.05, **P < 0.01, and ***P < 0.001.

Supplementary Table 1 Cell numbers and relative proportions of cell populations included in the CellChat analysis.

Supplementary Table 2 Human primer sequences used for quantitative real-time PCR. Forward and reverse primer sequences are presented in the 5′–3′ direction, and the corresponding literature sources are indicated by their PubMed identifiers (PMIDs).
